# Metastasizing dysgerminoma in an inland bearded dragon (*Pogona vitticeps*)

**DOI:** 10.1186/s12917-024-04368-2

**Published:** 2024-12-05

**Authors:** Silvana Schmidt-Ukaj, Rene Brunthaler, Michaela Gumpenberger

**Affiliations:** 1 Service for Birds and Reptiles, Clinical Centre for Small Animals, University of Veterinary Medicine, Vienna, Austria; 2 Institute for Pathology, University of Veterinary Medicine, Vienna, Austria; 3 Clinical Unit for Diagnostic Imaging, University of Veterinary Medicine, Vienna, Austria

**Keywords:** Lizard, Neoplasm, Ovarian neoplasms, Reptiles, Diagnostic imaging, CT

## Abstract

**Background:**

Malignant dysgerminomas are infrequently reported ovarian neoplasms in animals, especially in exotic pets (non-traditional companion animals [NTCAs]). In the few published case reports on reptilian species, examples are primarily postmortem without antemortem (clinical) assessment.

**Patient presentation:**

An adult, 13-year-old, spayed female inland bearded dragon (*Pogona vitticeps*) presented with lethargy, a right-sided head tilt, unilateral exophthalmos and ventrotemporal strabismus on the right eye. On examination, a palpable mass (approximately 3.5 cm in diameter) was detected within the mid coelomic cavity. Computed tomography revealed a retrobulbar swelling and lytic bone lesions affecting the right frontal bone and several vertebrae (T11, T13, and T14). Multiple nodules of soft tissue opacity were also detected within the lungs, liver, and coelomic fat bodies. Haematology revealed leukocytosis with heterophilia and toxic granulation of heterophils. On the basis of these results, differential diagnoses included disseminated abscesses, granulomas (e.g., due to mycobacteriosis) and neoplasms. The lizard was subsequently euthanized due to end-stage disease and a poor prognosis. Postmortem gross examination and histopathology revealed a primary ovarian dysgerminoma with evidence of widespread metastasis as well as localized tissue destruction affecting the right retrobulbar space and frontal bone, the spinal column, the lungs, the liver, and both coelomic fat bodies.

**Conclusions:**

This report describes a case of malignant dysgerminoma with widespread intraosseous and visceral metastases in a captive inland bearded dragon (*Pogona vitticeps*).

## Background

Neoplastic diseases in reptiles have traditionally been considered rare; however, with growing interest in exotic animal medicine and advancing diagnostic techniques, the number of identified neoplasms is constantly increasing. According to recent large cohort studies, the prevalence of neoplasms in lizard populations ranges between 2.6% and 22.9%, with hepatobiliary, hematopoietic and musculoskeletal systems, as well as skin and subcutis being frequently affected. Neoplasms of the reproductive tract are found in only 0.1–1.2% of lizards and adenocarcinomas are most commonly diagnosed [12, 20, 23, 35, 36].

This report describes a case of malignant dysgerminoma with widespread intraosseous and visceral metastases in a captive inland bearded dragon (*Pogona vitticeps*) [32].

## Case presentation

An adult, 13-year-old, spayed, female inland bearded dragon (*Pogona vitticeps*) presented at the Service for Birds and Reptiles at the Veterinary University Vienna after a gradual onset of lethargy, head tilt, exophthalmos and ventrotemporal strabismus on the right eye.

The lizard was housed in a glass vivarium (160 × 60 × 80 cm) at an average temperature of 30 °C. The owner did not provide any artificial UV light, but the bearded dragon was given occasional access to the balcony for sunbathing in summer, although the total amount of hours per day was unknown. The lizard was fed greens, fruits and insects with a multivitamin and calcium powder. The owner reported that the animal had previously undergone an ovariosalpingectomy at another veterinary clinic, followed by an explorative coeliotomy due to the presence of persistent ovarian remnants. In addition to the aforementioned clinical signs, the lizard exhibited dehydration, evidenced by enophthalmos of the left eye and increased skin wrinkling. The patient was in poor body condition (BCS 2/5) with significant muscle atrophy of the tail and pelvic muscles, and displayed generalized muscle tremors along with occasional dyspnoea. On coelomic palpation, a single, irregularly round, 3.5 cm diameter mass was detected within the mid-coelomic cavity.

Haematology and plasma biochemistry analysis revealed leukocytosis (with heterophilia and toxic granulation of heterophils) with no significant biochemical changes other than hyperglycaemia (Table 1).

**Table 1:** Haematology and plasma biochemistry parameters of an adult female inland bearded dragon (*Pogona vitticeps*) diagnosed with an ovarian dysgerminoma and metastatic disease

Parameter	Patient	Reference^a^ Mean (Range)
**Haematology**		
PCV (%)	29	30 (17–45)
WBC (10^3^/µL)	44	6.21 (1.45-19.0)
Heterophils (10^3^/µL)	42.24	2.09 (0.24–7.77)
Lymphocytes (10^3^/µL)	0.44	2.77 (0.29–11.3)
Monocytes (10^3^/µL)	1.32	0.25 (0.03–1.39)
**Plasma Biochemistry**		
Glucose (mg/dl)	570	202 (108–333)
Uric acid (mg/dl)	2.9	3.1 (0.5–9.8)
Protein, total (g/dl)	4.76	5.0 (3.0-8.1)
Bile acids (µmol/L)	14	x
Calcium (mg/dl)	12.8	11.9 (8.6–18)
Phosphorus (mg/dl)	3.9	4.4 (2.1–10.6)

^a^Klaphake E, Gibbons PM, Sladky KK, Carpenter JW. Reptiles, in Exotic Animal Formulary, 5th Edition, Saunders. 2017. pp 776

**Fig. 1:**
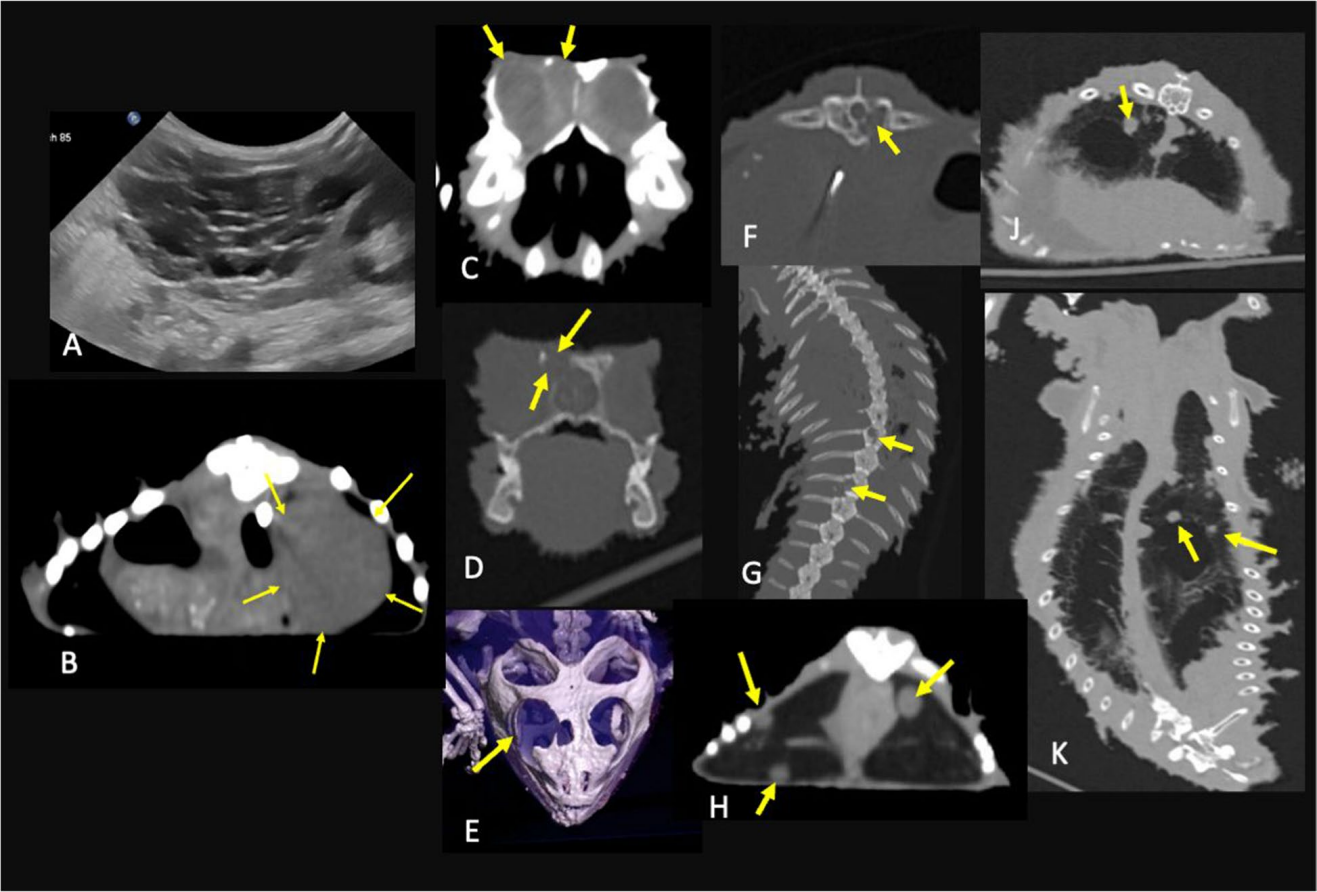
(**A**) Sonographic image of a cystic mass at the site of the removed ovary in an inland bearded dragon (*Pogona vitticeps*). (**B**) to (**K**) Computed tomography (CT) images of the head (**C**-**E**), spine (**F**, **G**), coelomic cavity (**B**, **H**), and lung (**J**, **K**) of an inland bearded dragon. (**B**, **C**, **H**) Soft tissue window; (**D**, **F**, **G**, **J**, **K**) Bone or modified bone window; and (**E**) three-dimensional model of the skull. (**B**, **C**, **D**, **F**, **H**, **J**) The images are in the transverse plane, whereas (**G**,** K**) the images are in the coronal plane. The arrows in (**B**) highlight the ovarian mass, which was subsequently diagnosed as dysgerminoma. Note that the cystic composition is better visualized via sonography (**A**). In (**C**) the retrobulbar soft tissue mass (arrows) of the right eye is visible, and in (**D**), frontal bone lysis (arrows) is visible. The extend of the frontal bone osteolysis and caudal displacement of the eye and the osseous scleral ring (arrow) are shown (**E**). Figure (**F**) and (**G**) highlight the lytic vertebrae. In (**F**), the arrow indicates the defect at T11 (T - thoracic vertebra), and in (**G**), the arrows indicate the lesions at T11 and T13/14. Multiple hyperdense nodules (arrows) were found in the fat body (**H**) and lung (**J**, **K**)

Ultrasound examination of the coelomic cavity revealed a 4 × 3.3 cm large multilobulated oval mass at the level of the aforementioned removed ovaries. The mass contained multiple septa and cysts and could not be further assigned to any organ system (Fig. 1). A subsequent computed tomography (CT) examination revealed lytic bone lesions affecting the right frontal bone with retrobulbar expansion, as well as osteolysis of thoracic vertebrae (T) (T11, and T13/T14). Multiple soft tissue nodules of various sizes could be found in the lungs, liver and fat bodies (Fig. 1). Based on imaging findings, the differential diagnoses included disseminated abscesses or granulomas (e.g., due to mycobacteriosis), or neoplastic disease.

The patient was euthanised due to a poor prognosis and the owner agreed to further investigations. Euthanasia was performed by intravenous injection with ketamine (100 mg/kg IV, Ketamidor^®^ 100 mg/ml) and xylazine (10 mg/kg IV, Sedaxylan^®^ 20 mg/ml), followed by pentobarbital into the ventral coccygeal vein (450 mg/kg IV, Release^®^ 300 mg/ml). Cardiac arrest was confirmed with Doppler ultrasonography.

Postmortem examination revealed a 3.5 cm diameter, partly solid, firm, ovoid, off-white-to-yellow coloured mass located at the site of the left ovary. Multiple embedded cysts were filled with clear fluid. The histopathological examination revealed a multilobular, highly cellular neoplasm that consisted of round to polygonal, approximately 4–14 μm long, regionally degenerated tumour cells with mostly indistinct cell borders. These cells did not form a true cluster and were loosely arranged in cords or broad sheets, occasionally separated by rather fine fibrovascular stroma. The tumour cells presented moderate anisocytosis and a sparse, pale, eosinophilic cytoplasm. The predominantly eccentric, occasionally hyperchromatic nuclei were round with one, rarely two prominent nucleoli and coarsely to finely granularly distributed chromatin and presented moderate anisokaryosis. Occasionally, multinucleated tumour cells and elevated mitotic figures (over 10 mitotic figures in 10 high-power fields (2.37 mm^2^)) were also observed. Individual areas of the tumour were necrotic or showed discrete haemorrhages. The mass was thus referred to as dysgerminoma.

**Fig. 2:**
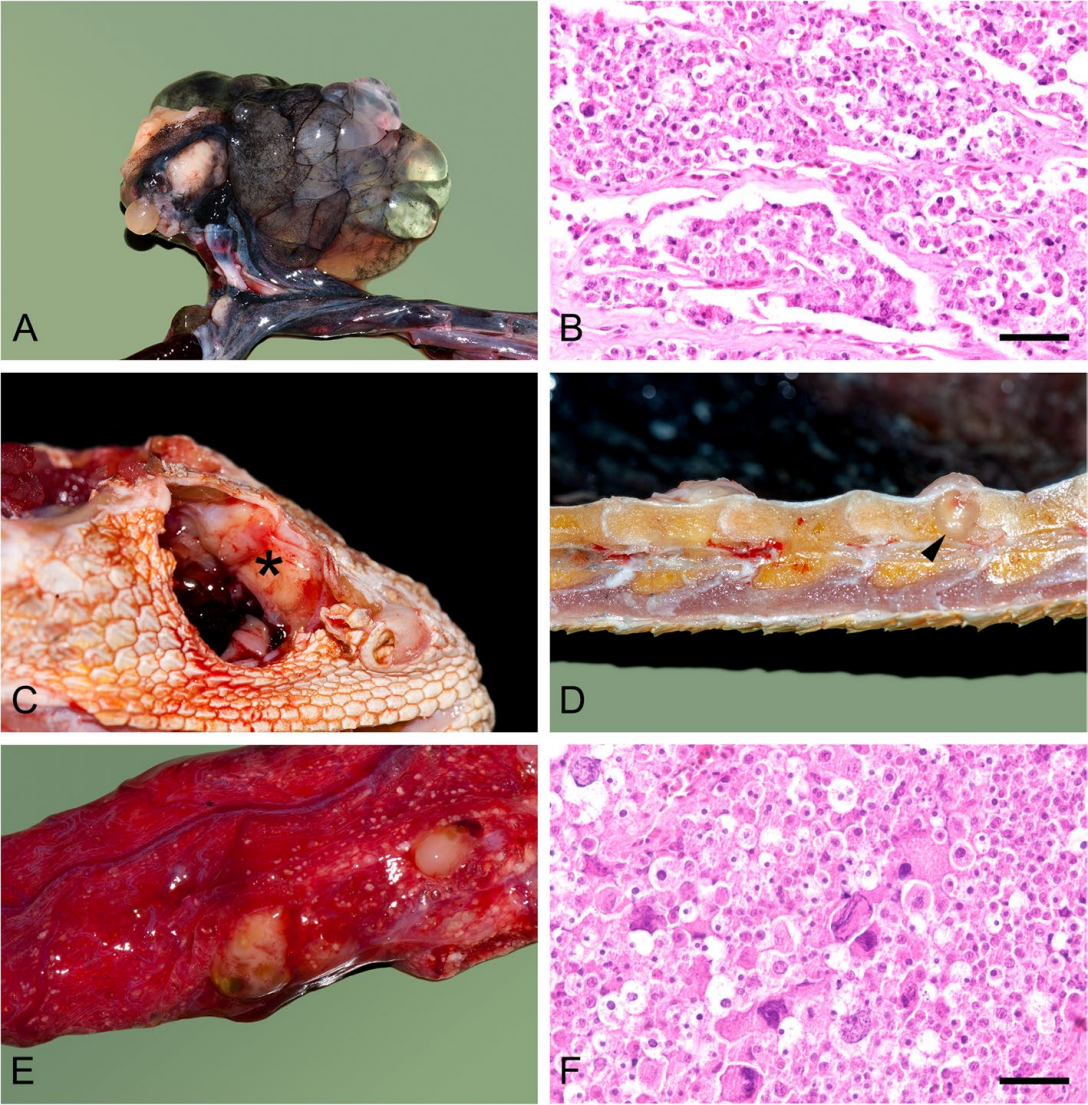
**A** A 3.5 cm diameter mass in the area of the left ovary, consisting partly of a firm, solid, whitish-yellowish mass and partly of multiple cysts. **B** Photomicrograph of a neoplasm of the left ovary with tumour cells arranged in cord-like formations and separated by fine fibrovascular stroma (H&E staining, scale bar = 40 μm). **C** Metastases of a dysgerminoma infiltrating the frontal bone in the right retrobulbar space (asterisk). **D** Longitudinal section of the spinal column with ventrolateral metastasis of the dysgerminoma infiltrating the vertebral body and simultaneously leading to a lytic bone lesion (arrowhead). **E** Multiple, raised, white‒yellowish, coarse nodules in the lung parenchyma, which histologically appear as metastases of the dysgerminoma. **F** Microscopy image of a dysgerminoma metastasis in the lung with solidly arranged tumour cells and individual multinucleated giant cells as well as mitosis (H&E staining, scale bar = 40 μm)

Multiple yellow‒grey masses were found in the right retrobulbar space infiltrating the frontal bone causing significant osteolysis. They were histologically identified as metastases of the primary coelomic tumour and considered the cause of the clinically observed low-grade right-sided exophthalmos. Similarly, greyish, coarse nodules consistent with dysgerminoma metastases were visible ventrolaterally on the spinal column (T11, T13, and T14), which infiltrated the bones and led to lytic bone lesions. In addition, multiple metastatic foci were found in the lungs, with yellowish nodules up to approximately 0.5 cm in size, and sporadically in the liver, with yellowish-white, spherical foci up to 1 cm in size. Finally, individual 0.5 cm large tumour metastases were also located in both fat pads (Fig. 2). There were no other abnormalities detected during the postmortem examination macroscopically or histologically.

## Discussion

Ovarian tumours can be classified on the basis of their cell origin into epithelial tumours, germ cell tumours and sex cord–stromal tumours. Germ cell tumours include dysgerminomas and teratomas [25].

In women, malignant dysgerminomas are typically reported under 30 years of age [37]. They can spread throughout the body via carcinomatosis (direct peritoneal spread) or through the blood and lymphatic system to areas such as the bone, lungs, liver, spinal cord and brain [2, 13, 31]. The incidence of ovarian cancer in dogs and cats is low because they often undergo early ovariohysterectomy. Dysgerminomas represent approximately 6–12% of canine ovarian neoplasms, and 10–30% of them metastasize [7, 26, 29]. Infiltration of neoplastic cells in the nervous system has been observed in only one dog with dysgerminoma [8]. Single cases of dysgerminomas have been reported in other animal species, such as birds, amphibians, and fish, including Chondrichthyes, but were mostly discovered on postmortem examinations and often without any previous clinical diagnosis [9, 17, 19, 21, 22, 33].

In reptiles, the first cases of ovarian dysgerminomas were reported by Frye et al. in 1988 [10] in two red-eared slider turtles (*Trachemys scripta elegans*) and by Machotka et al. in 1992 [24] in a snapping turtle (*Chelydra serpentina*). Diagnoses were made on postmortem examinations without any clinical evaluation or diagnostic imaging of the living patient. A case of malignant ovarian dysgerminoma was recently described in a leopard gecko (*Eublepharis macularius*), which was the first report of antemortem diagnosis and treatment in a lizard. The tumour was surgically removed, and the patient recovered successfully. The excised mass showed invasion into blood vessels on histopathology [40]. Further reports of dysgerminomas include Heckers´ 2017 conference abstract describing the neoplasm in a lizard [18] and a submission of the tumour from a green iguana (*Iguana iguana*) to the Registry of Tumours in Lower Animals (RTLA), Sterling, Virginia, USA. In these two cases, no further information was provided. In a recent study reviewing cases of neoplastic disease in reptiles in a veterinary teaching hospital, dysgerminomas were found in a corn snake (*Pantherophis guttatus*) and in an inland bearded dragon (*Pogona vitticeps*). In both cases complete surgical removal was curative [35].

Sonographic examinations and computed tomography are becoming increasingly available in exotic pet medicine. Both imaging tools are non-invasive and images can often be acquired very fast and without the need for sedation or general anaesthetic. For sonographic examinations, the lizards are manually restrained and scanned with a high-frequency transducer (7.5–12 MHz, linear or microconvex transducer) and an efficient amount of coupling gel. An experienced radiologist with knowledge of the species-specific anatomy is invaluable to perform an in-depth examination. One major advantage of sonography is the detailed demonstration of the inner architecture of soft tissues and the identification of even small amounts of fluid accumulation. For CT examination, smaller lizards are usually placed in a narrow plastic box without sedation and images are obtained within a few minutes. On CT, all organ systems can be visualized at the same time without superimposition, which is a major advantage in patients with multiple diseases. The application of contrast medium can further enhance soft tissue differentiation [3–5, 15, 16, 28].

In the presented case, we could differentiate lytic bone lesions of the skull and multiple vertebrae, define retrobulbar soft-tissue expansion, and detect multiple soft-tissue nodules in the lungs, liver and fat bodies as well as a coelomic cystic mass. Although CT can positively identify all affected organs, the definitive diagnosis of an ovarian neoplasm is reserved for histopathology. On gross pathology dysgerminomas present as solid masses with multiple additional cysts. Histological examination using standard haematoxylin and eosin standard staining, reveals a typical pattern of uniform tumour cells arranged in nests and often separated by fibrous septa. The neoplastic cells present with an eosinophilic cytoplasm, distinct cell membranes and rounded nuclei (Fig. 2.). However, this is not always the case, as there may be unusual morphology patterns. An additional immunohistochemical examination is a valuable tool for making a reliable diagnosis, whereby a combination of positive nuclear expression of SALL4 and OCT4 and positive membranous/cytoplasmic expression of CD117 and D2-40 in a tumour is specific for a dysgerminoma in humans [30]. Although some reports have evaluated immunohistochemical staining to confirm the diagnoses of dysgerminomas in exotic animals [9, 33], appropriate IHC protocols and cross-reactivity in reptiles are still lacking and further research is required. The ovarian dysgerminoma in the present case report was grossly and microscopically similar to other reported dysgerminomas [33].

Ovariosalpingectomy of pet reptiles is performed for the treatment of dystocia. But the ovarian tissue in lizards is often diffuse and tightly associated with the caudal vena cava and adrenal gland, which often makes complete ovariectomy challenging. If ovariectomy is incomplete, even small tissue residues may regrow, and folliculogenesis will resume [6]. This explains why the patient in the present case report underwent a second surgical procedures to remove remnant ovarian tissue following initial ovariosalpingectomy at an external veterinary clinic. It has been well reported in women [11, 39], dogs, cats [1, 34] and lizards [6], that this remaining tissue can initiate neoplastic development.

The recommended therapy of dysgerminomas in women is unilateral salpingo-oophorectomy with or without combined chemo- or radiotherapy [27]. In dogs, a combination of surgery, chemo- and radiotherapy lead to promising outcomes [14, 38]. However, in the current case these treatment options were not considered feasible due to widespread end-stage disease.

## Conclusion

This report represents a case of malignant dysgerminoma with widespread intraosseous and visceral metastases in a captive inland bearded dragon (*Pogona vitticeps*). Dysgerminomas are uncommonly reported neoplasms in reptile medicine. CT has been proven to be a useful and quick antemortem tool for evaluating the extent of disease at presentation as well as identifying unexpected further organ involvement. A definitive diagnosis of a dysgerminoma with multiple metastases was confirmed by histological examination.

## Data Availability

No datasets were generated or analysed during the current study.
